# Critical Hypercytokinemia in Sepsis and Septic Shock: Identifying Interleukin-6 Thresholds Beyond Which Mortality Risk Exceeded Survival Probability

**DOI:** 10.3390/jcm15031057

**Published:** 2026-01-28

**Authors:** Juan Carlos Ruiz-Rodríguez, Luis Chiscano-Camón, Adolf Ruiz-Sanmartin, Natalia Costa-Allué, Ivan Bajaña, Pablo Nicolas-Morales, Juliana Bastidas, Sergi Cantenys-Molina, Manuel Hernández-Gonzalez, Nieves Larrosa, Juan Jose González-López, Vicent Ribas, Ricard Ferrer

**Affiliations:** 1 Intensive Care Department, Vall d’Hebron University Hospital, Vall d’Hebron Barcelona Hospital Campus, 08035 Barcelona, Spain; 2 Shock, Organ Dysfunction and Resuscitation Research Group, Vall d’Hebron Research Institute (VHIR), Vall d’Hebron University Hospital, Vall d’Hebron Barcelona Hospital Campus, 08035 Barcelona, Spain; 3 Immunology Department, Vall d’Hebron University Hospital, Vall d’Hebron Barcelona Hospital Campus, 08035 Barcelona, Spain; 4 Microbiology Department, Vall d’Hebron University Hospital, Vall d’Hebron Barcelona Hospital Campus, 08035 Barcelona, Spain; nieves.larrosa@vallhebron.cat (N.L.);; 5 EHealth Unit, Eurecat, Centre Tecnològic de Catalunya, 08005 Barcelona, Spain

**Keywords:** hypercytokinemia, interleukin-6, severe sepsis, septic shock, cytokine hemoadsorption, immunomodulation

## Abstract

**Introduction**: Patients with extremely elevated IL-6 levels remain poorly characterized, and no specific plasma concentration has been established to reliably predict mortality or guide immunomodulatory interventions. We hypothesized that extreme hypercytokinemia is associated with increased mortality in sepsis. The primary objective was to identify, in patients with hyperinflammatory endotype, an IL-6 threshold associated with a significantly elevated risk of death. **Methods**: We conducted a retrospective, single-center observational study based on a historical cohort of adult patients with consecutive activation of the in-hospital sepsis code, a prospective and standardized institutional care pathway, at Vall d’Hebron University Hospital between July 2018 and December 2024. Patients fulfilling Sepsis-2 diagnostic criteria and criteria for severe sepsis or septic shock were eligible. Plasma interleukin-6 (IL-6) levels were routinely determined in all patients. The analysis included patients with complete clinical and laboratory data available in the study database. To identify the IL-6 threshold associated with critical risk of death, a cumulative conditional relative frequency analysis was performed. A quantile-based analysis was conducted using predefined intervals of 1000 pg/mL and 15,000 pg/mL. A multivariable logistic regression analysis was conducted to identify clinical and laboratory parameters independently associated with IL-6 > 15,000 pg/mL and outcome. Results are presented as odds ratios (ORs). Survival differences were assessed using Kaplan–Meier analysis. **Results**: Overall mortality was 31% in the 1669 patients analyzed. Median IL-6 concentration was 772 pg/mL (IQR: 164–8750 pg/mL) with significantly higher levels in non-survivors (2137 pg/mL, IQR: 267–34,758). A critical IL-6 cutoff of 14,930 pg/mL was identified, which was rounded to 15,000 pg/mL for clinical applicability. IL-6 > 15,000 pg/mL was associated with increased mortality (OR 2.22, 95% CI: 1.12–5.36). Kaplan–Meier analysis revealed significantly reduced survival in patients above this IL-6 threshold (*p* < 0.0001). **Conclusions**: In this cohort of patients with severe sepsis or septic shock, plasma IL-6 levels > 15,000 pg/mL defined a critical threshold beyond which mortality risk exceeded survival probability. Critical hypercytokinemia may serve as a clinically relevant biomarker to identify patients with sepsis and multiorgan dysfunction who could benefit from precision immunomodulatory therapies.

## 1. Introduction

Sepsis occurs when the initial appropriate response of the host to an infection becomes amplified and dysregulated, resulting in an imbalance between pro-inflammatory and anti-inflammatory responses [1,2,3]. An excess of pro-inflammatory cytokines can also lead to endothelial injury and systemic inflammatory response syndrome (SIRS) and so a tightly regulated equilibrium of the cytokine network is vital for eliminating pathogens and restricting tissue-damaging inflammation. In severe cases, sepsis can progress to shock, which exacerbates multiple organ dysfunction, ultimately resulting in death [4]. However, the inflammatory response to infection by innate immunity is usually controlled, localized, and protective [5]. The interaction between resistance (inflammatory response) and resilience (limiting inflammation by the adaptive immunity) is the key to survival, but in some circumstances not completely understood, this complex and delicate balance is lost, and sepsis syndrome may develop. In this process of dysregulated response, both the infected and distal organs may be injured, leading to a life-threatening clinical condition. Several studies [6,7,8,9] have suggested an association of interleukin-6 (IL-6) hypercytokinemia with organ dysfunction, response to treatment, and prognosis in sepsis. Patients with higher levels of IL-6 and interleukin-10 (IL-10) exhibit severe organ dysfunction and increased mortality [10].

In septic shock and multiorgan dysfunction that do not respond to standard treatment, hemoadsorption (HA) therapy may be considered as a rescue therapy. Cytokine HA may have a role as rescue therapy in a particular subgroup of patients with severe septic shock, multiorgan failure, and very high hypercytokinemia [11]. In this situation, blood purification techniques can effectively reduce the inflammatory process by rapidly and non-selectively targeting the cytokine storm, potentially improving survival benefit for the patient; however, it is important to note that currently, a universally accepted cytokine threshold to guide the initiation or discontinuation of hemoadsorption therapy has not yet been defined [12].

We hypothesized that extreme hypercytokinemia is associated with increased mortality in sepsis. The objective was to identify, in patients with sepsis or septic shock, a biologically meaningful threshold that defines a state of extreme hyperinflammation, herein referred to as “critical hypercytokinemia”, beyond which the probability of death exceeds that of survival. This threshold allows for characterizing a hyperinflammatory endotype associated with profound organ dysfunction and poor clinical trajectory.

## 2. Materials and Methods

### 2.1. Study Design, Inclusion and Exclusion Criteria

This was a retrospective, observational, single-center study based on a historical cohort of adult patients with consecutive activation of the in-hospital sepsis code at Vall d’Hebron University Hospital between July 2018 and December 2024. Sepsis code activation is part of a prospective, standardized institutional care pathway implemented across hospital departments.

The source population included all patients with sepsis code activation during the study period. For the present analysis, we included patients who fulfilled Sepsis-2 diagnostic criteria and for whom complete clinical and laboratory data for the variables of interest were available in the study database, including plasma interleukin-6 (IL-6) measurements.

Exclusion criteria were pregnancy, limitations of life-sustaining treatment, age <18 years, and incomplete or unavailable data in the study database.

### 2.2. Analyzed Data and Scores

The data collected included demographic variables, past medical history, clinical and analytical variables. The severity of the disease was evaluated with the Acute Physiology and Chronic Health Disease Classification System (APACHE) II [13] and Sequential Organ Failure Assessment (SOFA) scores [14]. Both scores were calculated using the worst parameters measured during the first 24 h of admission. The presence of septic shock or severe sepsis was defined according to the Sepsis 2 criteria [15]. ARDS was defined according to the Berlin definition criteria [16]. Data on the incidence of acute kidney injury (AKI) or failure, and the need for continuous renal replacement therapy (CRRT), were collected according to the latest Kidney Disease: Improving Global Outcomes (KDIGO) Clinical Practice Guideline criteria [17]. Length of ICU stay, and ICU and in-hospital mortality were registered. The study fulfilled the “Strengthening the reporting of observational studies in epidemiology (STROBE)” checklist for observational studies [18].

### 2.3. Cytokine Measurement

Circulating cytokine levels were analyzed on all patients, reflecting routine clinical practice. For that, blood samples were collected within the 12 first hours of evolution of sepsis or septic shock. The blood was centrifuged for 10 min at 1600× *g* within 1 h from withdrawal and serum samples were immediately processed. Circulating levels of IL-6 were tested using an electrochemiluminescence immunoassay: Elecsys^®^ IL-6 (Roche Diagnostics GmbH, Mannheim, Germany), from ROCHE.

### 2.4. Statistical Analysis

Data were summarized using *n* (%) for categorical variables and median (interquartile range, IQR) or mean (standard deviation, SD) for continuous variables. The normality of the data distribution was assessed using the Shapiro–Wilk test and Q–Q plots. Comparisons between groups were performed using Student’s *t* test for normally distributed variables or the Wilcoxon (Mann–Whitney U) test for non-normally distributed variables. Associations between categorical variables were examined using the χ^2^ test (chi-square test) or Fisher’s exact test, as appropriate.

To evaluate the association between the outcome and IL-6 as a continuous variable, a univariate analysis was performed using log10-transformed IL-6 values. To identify the IL-6 threshold associated with critical risk of death, a cumulative conditional relative frequency analysis was performed. This approach involved progressively evaluating IL-6 reference levels to determine the cutoff at which the mortality frequency exceeded 50%, interpreted as a clinically relevant inflection point. To ensure the robustness of this finding against data asymmetry, a quantile-based analysis was conducted using predefined intervals of 1000 pg/mL and 15,000 pg/mL. To assess independence from baseline severity, a multivariable sensitivity analysis was performed, adjusted for age and SOFA score, across a wide range of thresholds (200–60,000 pg/mL) to validate the independent prognostic value of the identified 15,000 pg/mL cutoff.

A multivariable logistic regression analysis was conducted to identify clinical and laboratory parameters independently associated with IL-6 > 15,000 pg/mL. Variables were selected based on univariate significance, clinical relevance, and the absence of multicollinearity (variance inflation factor, VIF < 5). Continuous predictors were transformed and standardized when appropriate to ensure model stability and interpretability. Internal validation was performed using bootstrap resampling, with 1000 iterations for the phenotype model, which included variables associated with IL-6 > 15,000 pg/mL, and 200 iterations for the outcome model, which included patients with IL-6 > 15,000 pg/mL, reflecting the size and complexity of each multivariable dataset. This approach allowed estimation of the stability of coefficients and model discrimination.

Results are presented as odds ratios (ORs) with 95% confidence intervals (CIs) and beta coefficients (βs) to facilitate model reproducibility. The discriminative performance was evaluated using receiver operating characteristic (ROC) curves and the corresponding area under the curve (AUC). Predictive performance was further quantified by calculating sensitivity, specificity, positive predictive value (PPV), and negative predictive value (NPV).

Finally, survival differences were assessed using Kaplan–Meier analysis and the log-rank test, with patients censored at 30 and 90 days to evaluate both short- and long-term clinical trajectories. All statistical analyses were performed using R software (version 4.1.0).

### 2.5. Ethics Statement

The study was approved by the Clinical Research Ethics Committee of Vall d’Hebron University Hospital [PR(AG)229/2024] with exemption from informed consent. The study compiled in full with the General Data Protection Regulation (GDPR) (Regulation (EU) 2016/679 [19]) and was performed in accordance with the ethical standards established in the 1964 Declaration of Helsinki and its later amendments. The committee accomplishes both in its composition and in the Standard Work Procedure (SWP) with the Best Clinical Practice (BCP) standards (CPMP/ICH/135/95) and with Royal Decree 1090/2015. The datasets used and analyzed during the current study are available from the corresponding author upon reasonable request. The authors declare that they have no competing interests. There was no fund reception. All authors were involved in providing care for the patient and they were all involved in writing and reviewing the manuscript. There were no acknowledgments; there were no contributions from individuals or organizations.

## 3. Results

### 3.1. Characteristics of the Study Population

A total of 1669 patients were included in the study [SOFA score: 6 (6); APACHE II: 20 (14); septic cardiomyopathy: 193 (11.7%); mechanical ventilation: 493 (29.5%)]. Of these, 1150 (69%) survived, while 519 (31%) died. Table 1a summarizes the population characteristics and Table 1b summarizes the microbiological characteristics of the study population.

### 3.2. Cytokine Analysis

**(1)**  **IL-6 levels in the overall study population and differences between survivors and non-survivors.**

Among the 1669 patients analyzed, the median IL-6 concentration was 772 pg/mL (IQR: 164–8750 pg/mL). IL-6 concentrations were significantly elevated in non-survivors (2137 pg/mL, IQR: 267–34,758) compared to survivors (552 pg/mL, IQR: 138–4656), (*p* < 0.001).

To confirm the robustness of the association between IL-6 and mortality independent of specific thresholds, a logistic regression analysis was performed using log10-transformed IL-6 values. The model demonstrated a highly significant continuous association, with mortality risk increasing by approximately 49% for every 10-fold increase in IL-6 concentration (OR 1.49; 95% CI 1.36–1.63; *p* < 0.001).

**(2)**  **Mortality associated with IL-6 levels thresholds**

**Figure 1:**
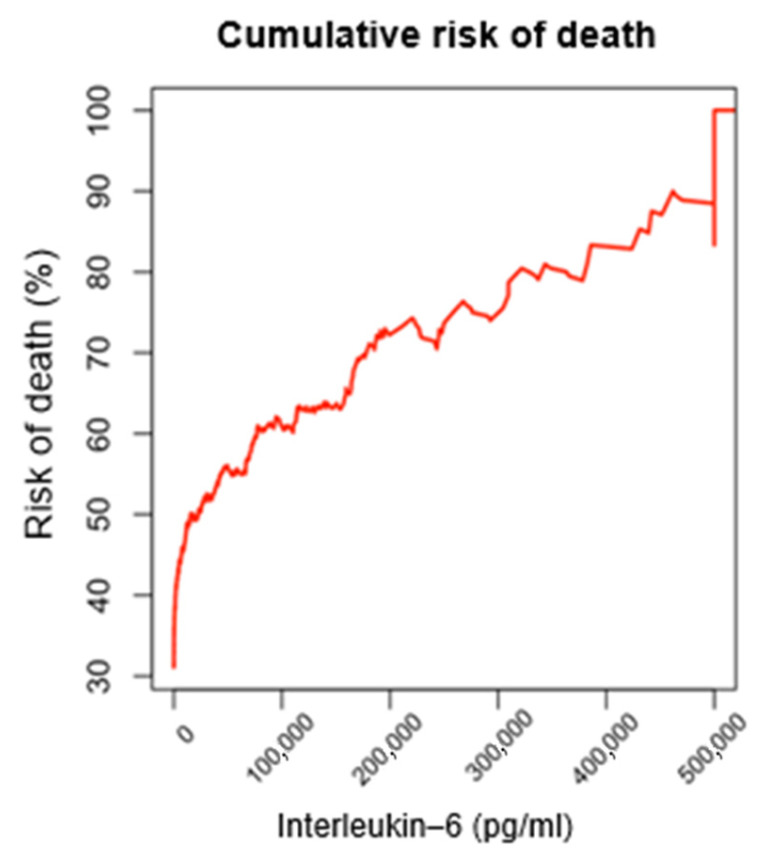
Cumulative mortality probability of the study population by Interleukin-6 plasmatic concentration.

A cumulative conditional relative frequency analysis was performed based on IL-6 levels considering the accumulation of survivor and non-survivor patients to identify survival patterns associated with these levels. The analysis established several IL-6 thresholds, identifying 14,930 pg/mL (95% CI: 8640–22,289 pg/mL) as the point at which mortality probability exceeds 50%, with 20% of patients above this level. This finding indicates that, beyond this threshold, the probability of survival begins to decrease significantly. Mortality probability increased to over 60% above 74,238 pg/mL (95% CI: 70,059–83,708 pg/mL), and reached 70% above 178,693 pg/mL (95% CI: 168,705–182,355 pg/mL). Notably, all patients with IL-6 ≥ 500,000 pg/mL (0.72% of the cohort) died (Figure 1, Table 2). For ease of clinical applicability and to ensure consistency across subsequent analyses, we rounded this value to 15,000 pg/mL for all further analyses.

**Figure 2:**
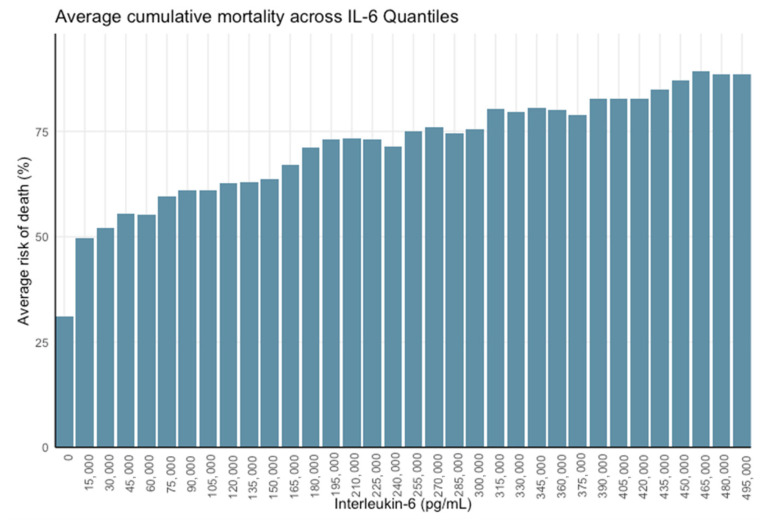
Mortality probability by 15,000 pg/mL IL-6 quantiles.

**Figure 3:**
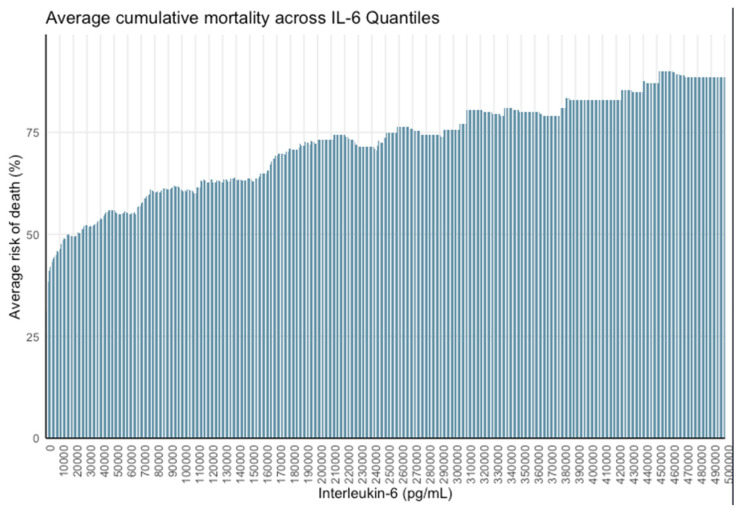
Mortality probability by 1000 pg/mL IL-6 quantiles.

Analysis using 15,000 pg/mL quantiles demonstrated a marked decrease in mortality below this threshold, with the most significant shift occurring between IL-6 < 15,000 pg/mL and ≥15,000 pg/mL. Finer stratification at 1000 pg/mL intervals confirmed the steepest mortality gradient within the 1.5–15,000 pg/mL range, indicating that survival probability increases most significantly below this threshold (Figure 2 and Figure 3).

A receiver operating characteristic (ROC) curve analysis identified 15,000 pg/mL as a relevant threshold for interleukin-6 (IL-6), beyond which the probability of mortality exceeds 50% (AUC = 0.62; specificity = 0.85, sensitivity = 0.34, *p* < 0.001). The Hosmer–Lemeshow goodness-of-fit test yielded a *p*-value of 0.12, indicating no significant evidence of lack of fit and suggesting an adequate agreement between predicted and observed outcomes.

IL-6 concentrations >15,000 pg/mL were associated with increased mortality (OR 2.22, 95% CI: 1.12–5.36), indicating a two- to threefold increase in the risk of death compared to patients with lower IL-6 levels. This cutoff was not established to optimize overall classification of survivors and non-survivors, but rather derived from a quantile-based analysis aimed at identifying the level at which mortality risk becomes predominant.

To account for baseline severity in this critically ill population, we performed an additional multivariable analysis adjusted for age and SOFA score. Univariate analysis confirmed their strong prognostic value: SOFA score (OR 1.27; 95% CI 1.23–1.31; *p* < 0.001) and age (OR 1.32; 95% CI 1.18–1.47; *p* < 0.001) were both highly significant predictors. The variance inflation factor (VIF) was assessed to confirm parameter independence and rule out multicollinearity (VIF < 5). Evaluation of model performance metrics across thresholds revealed stability in predictive power (AUC 0.75–0.76; sensitivity 0.71–0.82; specificity 0.56–0.66), largely driven by the strong prognostic weight of these severity scores; however, the 15,000 pg/mL threshold contributed to the maximal observed AUC (0.76) with a balanced sensitivity (0.77) and specificity (0.61). While log transformation confirmed a continuous association (OR 1.49; 95% CI 1.36–1.63; *p* < 0.001), our sensitivity analysis revealed that thresholds <15,000 pg/mL failed as independent predictors. The 15,000 pg/mL cutoff emerged as the specific inflection point (OR 1.35; 95% CI 1.02–1.80; *p* < 0.05) that remains significant independent of standard clinical scores, defining the distinct ”critical hypercytokinemia” phenotype.

**(3)**  **IL-6 >15,000 pg/mL as a defining threshold for hyperinflammatory endotype**

Table 3 shows a comparative analysis between patients with IL-6 levels > 15,000 pg/mL (n = 347) and those with IL-6 ≤ 15,000 pg/mL (n = 1322). The patients with IL-6 levels > 15,000 pg/mL showed more inflammation, severity and multiorgan dysfunction. The IL-6 > 15,000 pg/mL group also demonstrated higher rates of positive blood cultures, vasoactive support, renal replacement therapy, mechanical ventilation, and immunosuppression. These findings indicate that IL-6 > 15,000 pg/mL defines a subgroup with profound immunological dysregulation, greater disease severity, and a significantly worse clinical trajectory.

A multivariate analysis was conducted to identify clinical and laboratory parameters independently associated with the level IL6 > 15,000 pg/mL. The variance inflation factor (VIF) was assessed to confirm parameter independence and rule out multicollinearity (VIF < 5). Internal validation was performed using 1000 bootstrap resamples to estimate the stability of regression coefficients and model discrimination. The final model demonstrated excellent discrimination and robustness (AUC 0.91; Sensitivity 0.83; Specificity 0.85; PPV 0.60; NPV 0.95; *p* < 0.001); Intercept (−2.45). Bootstrap validation confirmed model stability, with 95% CI for AUC (95% CI 0.90–0.91) and consistent estimates for regression coefficients. The main associated variables were higher procalcitonin concentrations, vasoactive support, higher SOFA and increased lactate levels, and strong inverse association was observed with neutrophil and lymphocyte count. The results are shown in Table 4.

**(4)**  **Prognostic stratification within the IL-6 >15,000 pg/mL subgroup: clinical differences and independent predictors of mortality**
  **(4.1)**  **Differential clinical characteristics between survivors and non-survivors with IL-6 Levels >15,000 pg/mL**
    Survivors and non-survivors among patients with IL-6 levels exceeding 15,000 pg/mL exhibited distinct clinical profiles, with non-survivors showing a markedly more severe disease presentation (Table 5). Non-survivors exhibited significantly more severity and organ dysfunction. These findings confirm that within the IL-6 > 15,000 subgroup, mortality is closely linked to profound immune dysregulation, multiorgan failure, and advanced critical illness.
  **(4.2)**  **Independent Predictors of Mortality in patients with IL-6 Levels >15,000 pg/mL**
    A multivariate analysis was conducted to identify parameters independently associated with mortality in patients with IL-6 > 15,000. The variance inflation factor (VIF) was assessed to confirm parameter independence and rule out multicollinearity (VIF < 5). The final model demonstrated excellent discrimination (AUC 0.91; Sensitivity 0.85; Specificity 0.77; PPV 0.79; NPV 0.84; *p* < 0.05); Intercept (−0.53). Bootstrap validation confirmed model stability, with 95% CI for AUC (95% CI 0.88–0.91) and consistent estimates for regression coefficients. Among the predictors, SOFA score and immunosuppression were strongly and independently associated with the outcome (Table 6).
**(5)**  **Survival Analysis for Patients with IL-6 > 15,000 pg/mL**

**Figure 4:**
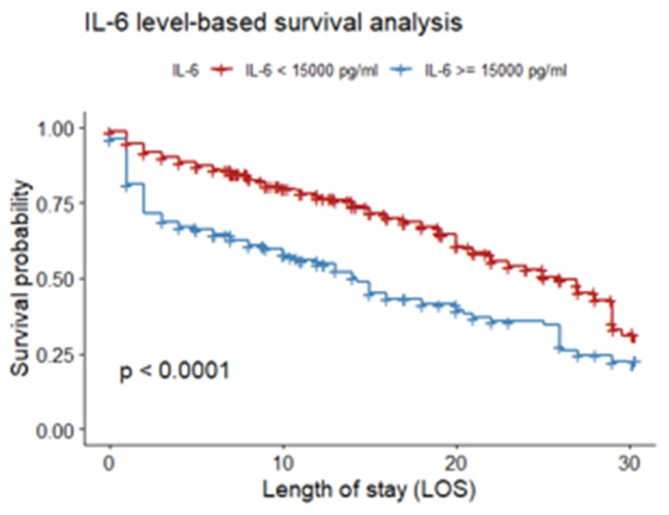
Survival analysis by IL-6 plasmatic concentration up to day 30.

**Figure 5:**
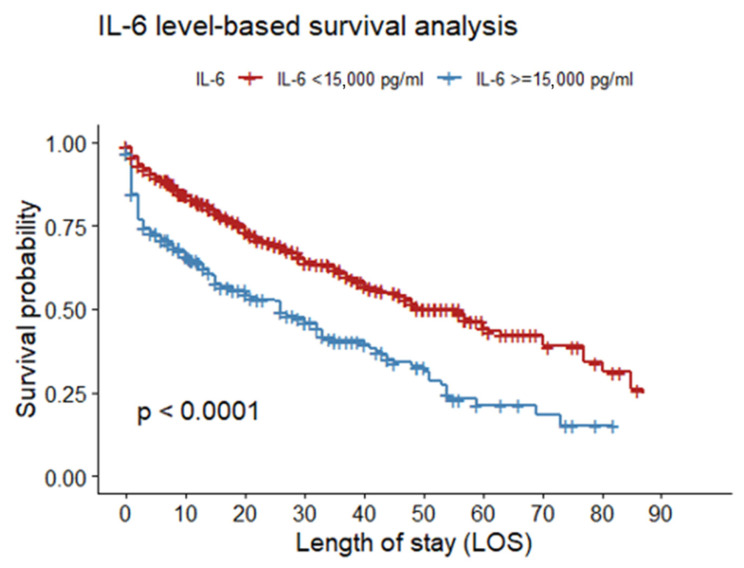
Survival analysis by IL-6 plasmatic concentration up to day 90.

The Kaplan–Meier survival curve was used to evaluate the prognostic significance of critical hypercytokinemia, defined as IL-6 levels exceeding 15,000 pg/mL. The analysis revealed a statistically significant reduction in survival probability in this group compared to patients with IL-6 ≤ 15,000 pg/mL (log-rank test, *p* < 0.001). The curve demonstrates a marked and rapid decline in survival in the early days of hospitalization, indicating high early mortality in this cohort (Figure 4 and Figure 5).

## 4. Discussion

In this study involving a large cohort of patients with severe sepsis and septic shock, a plasma IL-6 concentration exceeding 15,000 pg/mL was associated with an increased probability of death. Although the statistical analysis identified an optimal cutoff value of 14,930 pg/mL, we chose to round this figure to 15,000 pg/mL for subsequent analyses and clinical interpretation. This minor adjustment is unlikely to meaningfully impact predictive performance but enhances clarity, usability, and real-world applicability at the bedside. Our findings highlight IL-6 as a robust biomarker for identifying a hyperinflammatory state characterized by multiorgan dysfunction and high risk of adverse outcomes in critically ill patients. Importantly, this result carries two key implications: first, IL-6 levels above 15,000 pg/mL may serve as a clinically relevant threshold to define extreme or critical hypercytokinemia; and second, it may function as a precision medicine biomarker to identify patients most likely to benefit from immunomodulatory interventions, such as cytokine hemoadsorption therapies.

The aim of our study was not to develop a predictive model, but to identify a threshold that reflects a state of extreme hyperinflammation, which is physiopathologically linked to greater disease severity and consequently higher mortality risk. Therefore, the reported AUC (0.62) is not intended as a measure of predictive performance, but rather as statistical confirmation that the association between extreme IL-6 levels and mortality is not due to chance.

The identified IL-6 threshold of 15,000 pg/mL should not be interpreted merely as a numerical cutoff, but as a marker of a qualitatively distinct clinical state. Patients exceeding this level exhibited profound immune dysregulation, severe circulatory and respiratory failure, and a markedly increased need for advanced organ support, including vasoactive therapy, mechanical ventilation, and renal replacement therapy. Crossing this threshold appears to reflect a loss of effective inflammatory control, beyond which standard therapies are frequently insufficient.

Elevated IL-6 levels have been consistently associated with increased mortality in patients with septic shock. However, no universally accepted plasma threshold has been established that reliably correlates with mortality and could support clinical decision-making in critically ill patients. Previous studies have consistently demonstrated associations between elevated IL-6 levels and adverse outcomes in sepsis, often using continuous analyses or relatively low cutoff values. Our findings complement this body of evidence by identifying a high-level inflection point at which mortality becomes the predominant outcome. This perspective shifts the focus from incremental risk estimation to the delineation of a biologically distinct hyperinflammatory endotype with potential therapeutic implications.

Kellum et al. [20] investigated 120 patients with sepsis, including 60 with septic shock, and reported median IL-6 levels of 1200 pg/mL (IQR 800–2000 pg/mL) in non-survivors compared to 550 pg/mL (IQR 300–1000 pg/mL) in survivors. These findings suggested an association between elevated IL-6 and poor outcomes, reinforcing the notion that higher cytokine levels reflect the severity of the systemic inflammatory response. However, limitations in this study include its modest sample size, single-center design, and potential variability in IL-6 assays and measurement timing.

Pierrakos et al. [21] conducted a comprehensive review of over 600 septic patients across multiple cohorts. Despite heterogeneity in infection sources (e.g., pneumonia, intra-abdominal sepsis) and immune status, the authors found that patients with more severe septic shock frequently exhibited IL-6 values exceeding 5000 pg/mL. While specific cutoff thresholds varied, the review consistently underscored the prognostic relevance of elevated IL-6 across diverse septic populations. Yet, the heterogeneity of included studies, differences in patient selection, IL-6 measurement techniques, and sepsis severity definitions remain key limitations.

Molano-Franco et al. [22], in a systematic review and meta-analysis, assessed baseline IL-6 levels for predicting 28–30-day mortality in critically ill adult patients with sepsis. Although IL-6 levels were generally higher in non-survivors, the pooled effect size was clinically negligible (e.g., OR 1.02, 95% CI 1.01–1.03), suggesting limited predictive utility when adjusted for covariates such as age and severity scores. Reported median IL-6 values varied widely, typically between 100 and 1000 pg/mL.

Chousterman et al. [23] explored the pathophysiological mechanisms driving cytokine release across various forms of sepsis, including viral-induced shock. They reported that IL-6 levels >1000 pg/mL were frequently associated with more severe organ dysfunction, higher rates of acute respiratory distress syndrome, and increased mortality. However, the narrative nature of the review and reliance on heterogeneous data limited standardized threshold definitions and control for confounding variables.

In our series, IL-6 levels exceeding 15,000 pg/mL defined a distinct subgroup of patients with severe organ dysfunction, hyperinflammation, and markedly elevated mortality. Non-survivors showed significantly higher APACHE II, SOFA scores, lactate, proADM, and ferritin levels, worse respiratory parameters, more profound coagulation disturbances and thrombocytopenia, and greater need for vasopressors and renal replacement therapy.

Although IL-6 elevation is very common in sepsis, no consensus definition of hypercytokinemia currently exists. For example, Kobe et al. [24], in a small study with only seven sepsis patients, propose defining severe hypercytokinemia as IL-6 ≥1000–5000 pg/mL combined with systemic inflammatory response syndrome criteria or SOFA scores. However, our findings from a substantially larger cohort do not support this definition.

We consider it critical to define hypercytokinemia and establish clinically meaningful thresholds at which mortality risk significantly increases. This is particularly relevant as the cytokine storm underpins the pathophysiological rationale for hemoadsorption and immunomodulatory therapies, positioning IL-6 as a potentially useful biomarker. In our study, IL-6 levels > 15,000 pg/mL consistently identified a patient subgroup with more severe organ dysfunction, hyperinflammation, and a markedly elevated mortality risk.

Multiple complementary statistical approaches were intentionally employed in this study. These analyses were not designed to optimize individual-level mortality prediction, but rather to demonstrate the robustness and biological consistency of the identified threshold across distribution-sensitive and distribution-insensitive methods. The persistence of the association after adjustment for age and SOFA score supports the interpretation of IL-6 >15,000 pg/mL as an independent marker of a critical hyperinflammatory state rather than a mere surrogate of baseline severity.

Based on these findings, we propose the term **“critical hypercytokinemia”** to define IL-6 levels >15,000 pg/mL, representing a threshold where mortality risk surpasses survival probability in sepsis. Clinically, this threshold may signal when conventional therapies (e.g., antibiotics, fluids) become insufficient, and when advanced interventions such as cytokine hemoadsorption or targeted immunotherapies could be urgently required. From a research perspective, it offers a biomarker-defined framework to investigate immunomodulatory strategies in severe hyperinflammation. From a clinical perspective, the identification of critical hypercytokinemia may help to stratify patients who are unlikely to recover with standard care alone and provides a biologically based threshold that could be helpful for patient selection in future intervention trials evaluating precision immunomodulatory strategies.

This study has several limitations. First, this was a single-center retrospective study, and the analysis was restricted to patients with complete data availability in the study database, which may limit generalizability. Second, although IL-6 determination is routinely performed in all patients with sepsis code activation, the analysis was restricted to those patients with complete data availability in the study database, which may have introduced selection bias and reduced the final sample size. Third, patients were recruited from different clinical settings, including the emergency department, hospital wards, and the intensive care unit, which differ in monitoring intensity, therapeutic resources, and timing of interventions. This heterogeneity may have influenced both IL-6 levels and clinical outcomes and could be considered a potential confounding factor. However, it reflects the real-world application of a hospital-wide sepsis code designed to identify and manage sepsis across diverse care environments. Fourth, in our institutional protocol, plasma IL-6 sampling is performed at the time of in-hospital sepsis code activation, which is intended to occur early in the clinical course, before or shortly after initiation of sepsis-directed therapies. Therefore, IL-6 levels primarily reflect the initial inflammatory response rather than the effect of subsequent interventions. Information on therapies administered before or at the time of IL-6 sampling, including corticosteroids, immunomodulatory agents, or extracorporeal cytokine removal techniques, was not systematically available. Although IL-6 was measured at the time of sepsis code activation, a few probable early therapeutic interventions may have influenced cytokine levels and represent a potential source of residual confounding. Fifth, the ROC curve was used as a statistical validation tool to exclude chance as an explanation for the observed association. Although the model does not provide optimal discrimination across the full IL-6 range, the chosen threshold effectively excludes most survivors and isolates a subgroup with markedly elevated IL-6 levels and substantially increased mortality risk. Such extreme IL-6 values are uncommon among survivors, highlighting the threshold’s discriminative value in the upper distribution tail. Both survivors and non-survivors can exhibit low IL-6 levels; the clinical relevance of this biomarker resides in its capacity to detect severe hypercytokinemia. Finally, as it reflects real-world clinical practice, we cannot guarantee that all patients were at comparable inflammatory phases, introducing potential variability in biomarker interpretation.

## 5. Conclusions

In this study, in patients with sepsis and septic shock, we identified that IL-6 levels exceeding 15,000 pg/mL define a subgroup of patients with extreme hyperinflammation, severe organ dysfunction, and a markedly elevated risk of mortality. We propose the phrase “critical hypercytokinemia” to characterize this extreme inflammatory state, which has both clinical and research implications. Clinically, it may serve as a biomarker precision medicine tool to identify patients who are unlikely to respond to conventional therapies and who may benefit from advanced immunomodulatory approaches. From a research standpoint, this threshold offers a framework to stratify patients for clinical trials investigating novel interventions targeting severe hyperinflammation.

## Figures and Tables

**Table 1:** (**a**) Characteristics of the study population. The data have been expressed as “n” (%) if they are categorical and as median (interquartile range) or mean (standard deviation) if they are quantitative. APACHE: Acute Physiology and Chronic Health Evaluation; CRRT: continuous renal replacement therapy; ICU: intensive care unit; IL: interleukin; IMV: invasive mechanical ventilation; PCT: procalcitonin; SOFA: Sequential Organ Failure Assessment. (**b**) Microbiological characteristics of the study population.

(**a**)		
	**N = 1669**	
**Age**, [years, mean (SD)]	65.9 (16.8)	
**Gender** [n (%)]	Female	631 (37.8)
	Male	1038 (62.2)
**APACHE II**, mean (SD)	20.4 (9.15)	
**SOFA**, median (IQR)	6 (4–10)	
**ICU admission**, [n (%)]	703 (43)	
**Severe sepsis**, [n (%)]	778 (46.6)	
	Mortality [n (%)]	163 (21)
**Septic shock**, [n (%)]	891 (53.4)	
	Mortality [n (%)]	356 (40)
**Septic cardiomyopathy**, [n (%)]	193 (11.7)	
**Positive blood culture**, [n (%)]	679 (40.8)	
**Infection source**, [n (%)]	Respiratory	548 (32.8)
	Abdominal	460 (27.6)
	Urinary tract	377 (22.6)
	Soft tissue	98 (5.8)
	Primary bacteriemia	49 (2.9)
	Catheter-related bacteremia	46 (2.7)
	Central nervous system	7 (0.4)
**Analytic parameters**	Leukocyte [(/mm^3^), median (IQR)]	11,320 (5470–17,640)
	Neutrophil [(/mm^3^), median (IQR)]	9500 (4000–15,200)
	PCT [(ng/mL), median (IQR)]	3.43 (0.64–19.7)
	IL-6 [(pg/mL), median (IQR)]	772 (164–8750)
	Lactate [(mg/dL), median (IQR)]	2.4 (1.5–4.3)
	Platelet count [(×10^9^/L), mean (SD)]	179,000 (106,000–269,000)
	proADM [(nmol/L), median (IQR)]	3.48 (1.81–7.77)
	INR, mean (SD)	1.24 (1.1–1.46)
**IL-6** [(pg/mL), median (IQR)]	All population	772 (164–8750)
	Alive	552 (138–4656)
	Dead	2137 (267–34,758)
**Dobutamine**, [n (%)]	145 (8.73)	
**Vasopressin**, [n (%)]	214 (13.9)	
**IMV**, [n (%)]	493 (29.5)	
**IMV days**, median (IQR)	2 (0–13)	
**CRRT**, [n (%)]	244 (14.6)	
**Outcomes**	**Hospital stay** [days, m(SD)]	13 (5–26)
	**Hospital mortality** [n (%)]	519 (31)
(**b**)		
**Blood cultures** ** [n(%)] **		**1663 (99.64)**
**Positive blood cultures** [n (%)]		679 (40.82)
**Microorganisms**		
*Escherichia coli* [n (%)]		193 (11.56)
*Klebsiella pneumoniae* [n (%)]		106 (6.35)
*Staphylococcus aureus* [n (%)]		46 (2.76)
*Pseudomonas aeruginosa* [n (%)]		43(2.58)
*Staphylococcus epidermidis* [n (%)]		37(2.22)
*Proteus mirabilis* [n (%)]		27 (1.62)
*Enterococcus faecium* [n (%)]		23 (1.38)
*Streptococcus pneumoniae* [n (%)]		18 (1.08)
*Staphylococcus hominis* [n (%)]		16 (0.96)
*Enterobacter cloacae* [n (%)]		13 (0.78)
*Klebsiella oxytoca* [n (%)]		10 (0.6)
*Enterococcus faecalis* [n (%)]		9 (0.54)
*Staphylococcus haemolyticus* [n (%)]		9 (0.54)
*Streptococcus pyogenes* [n (%)]		9 (0.54)
*Bacteroides fragilis* [n (%)]		8 (0.48)
*Enterobacter aerogenes* [n (%)]		7 (0.42)
*Serratia marcescens* [n (%)]		7 (0.42)
*Streptococcus anginosus* [n (%)]		7 (0.42)
*Candida glabrata* [n (%)]		4 (0.24)
*Citrobacter koseri* [n (%)]		4 (0.24)
*Streptococcus constellatus* [n (%)]		4 (0.24)
*Streptococcus dysgalactiae* [n (%)]		4 (0.24)
*Bacteroides thetaiotaomicron* [n (%)]		3 (0.18)
*Morganella morganii* [n (%)]		3 (0.18)
*Parvimonas micra* [n (%)]		3 (0.18)
*Staphylococcus capitis* [n (%)]		3 (0.18)
*Streptococcus grup mitis* [n (%)]		3 (0.18)
*Bacteroides uniformis* [n (%)]		2 (0.12)
*Candida albicans* [n (%)]		2 (0.12)
*Candida parapsilosis* [n (%)]		2 (0.12)
*Clostridium tertium* [n (%)]		2 (0.12)
*Corynebacterium* sp. [n (%)]		2 (0.12)
*Eggerthella lenta* [n (%)]		2 (0.12)
*Haemophilus influenzae* [n (%)]		2 (0.12)
*Klebsiella* sp. [n (%)]		2 (0.12)
*Listeria monocytogenes* [n (%)]		2 (0.12)
*Streptococcus agalactiae* [n (%)]		2 (0.12)
*Streptococcus gallolyticus-pasteurianus* [n (%)]		2 (0.12)
*Streptococcus mitis* [n (%)]		2 (0.12)
*Streptococcus parasanguinis* [n (%)]		2 (0.12)
*Abiotrophia defectiva* [n (%)]		1 (0.06)
*Actinomyces viscosus* [n (%)]		1 (0.06)
*Bacillus cereus* [n (%)]		1 (0.06)
*Bacillus firmus* [n (%)]		1 (0.06)
*Bacillus* sp. [n (%)]		1 (0.06)
*Bacteroides* sp. [n (%)]		1 (0.06)
*Branhamella catarrhalis* [n (%)]		1 (0.06)
*Burkholderia contaminans* [n (%)]		1 (0.06)
*Campylobacter jejuni* [n (%)]		1 (0.06)
*Candida guilliermondii* [n (%)]		1 (0.06)
*Candida lusitaniae* [n (%)]		1 (0.06)
*Candida tropicalis* [n (%)]		1 (0.06)
*Chryseobacterium gleum* [n (%)]		1 (0.06)
*Clostridium baratii* [n (%)]		1 (0.06)
*Clostridium paraputrificum* [n (%)]		1 (0.06)
*Clostridium septicum* [n (%)]		1 (0.06)
*Enterococcus casseliflavus* [n (%)]		1 (0.06)
*Fusobacterium necrophorum* [n (%)]		1 (0.06)
*Lactobacillus* sp. [n (%)]		1 (0.06)
*Proteus vulgaris* [n (%)]		1 (0.06)
*Providencia rettgeri* [n (%)]		1 (0.06)
*Pseudomonas* sp. [n (%)]		1 (0.06)
*Raoultella ornithinolytica* [n (%)]		1 (0.06)
*Rhodococcus* sp. [n (%)]		1 (0.06)
*Salmonella enteritidis* [n (%)]		1 (0.06)
*Salmonella* sp. [n (%)]		1 (0.06)
*Serratia liquefaciens* [n (%)]		1 (0.06)
*Staphylococcus lugdunensis* [n (%)]		1 (0.06)
*Staphylococcus simulans* [n (%)]		1 (0.06)
*Stenotrophomonas maltophilia* [n (%)]		1 (0.06)
*Streptococcus gallolyticus-gallolyticus* [n (%)]		1 (0.06)
*Streptococcus gordonii* [n (%)]		1 (0.06)
*Streptococcus grup salivarius* [n (%)]		1 (0.06)
*Streptococcus intermedius* [n (%)]		1 (0.06)

**Table 2:** Description of the observed mortality probability above the identified IL6 threshold.

Mortality Probability (%)	IL-6 (pg/mL)	CI (95%)	% Patients Above the Threshold
30	1.5	1.5–118.5	100%
40	1427	456.9–4755	42.84%
50	14,930	8640–22,289	20%
60	74,238	70,059–83,708	10%
70	178,693	168,705–182,355	5%
80	321,726	170,549–500,000	3%
90	500,000	382,573–2,652,000	2%

**Table 3:** Clinical characteristics and outcomes of patients with IL-6 levels >15,000 pg/mL. AKI: acute kidney injury, APACHE II: Acute Physiology and Chronic Health Evaluation II, CRP: C-reactive protein, CRRT: continuous renal replacement therapy, HFNC: high-flow nasal cannula, IgA: immunoglobulin A, IgG: immunoglobulin G, IgM: immunoglobulin M, IL-6: interleukin-6, IMV: invasive mechanical ventilation, PaFi (PaFiO_2_): ratio of arterial oxygen partial pressure to fraction of inspired oxygen, PCT: procalcitonin, proADM: proadrenomedullin, SaFi: ratio of arterial oxygen saturation to fraction of inspired oxygen, SOFA: Sequential Organ Failure Assessment.

	IL-6 ≤ 15,000 pg/mL (n = 1322)	IL-6 Levels > 15,000 pg/mL (n = 347)	*p*-Value
**Age** (years), mean (SD)	68 (16.64)	66 (17.07)	NS
**Gender** (female), n	511 (38.7)	120 (34.6)	NS
**SOFA**, median (IQR)	6 (3–9)	10 (7–13)	<0.001
**APACHE II**, mean (SD)	19 (8.776)	24 (9.367)	<0.001
**Severe sepsis**, n (%)	722 (54.6)	56 (16.1)	<0.001
**Septic shock**, n (%)	600 (45.4)	291 (83.9)	<0.001
**Leukocytes** (/mm^3^), median (IQR)	13,000 (7790–18,760)	3835 (124–10,310)	<0.001
**Neutrophils** (/mm^3^), median (IQR)	1080 (5900–16,100)	2300 (400–7125)	<0.001
**Lymphocytes** (/mm^3^), median (IQR)	650 (400–120)	20 (10–500)	<0.001
**INR**, median (IQR)	1.20 (1.09–1.42)	1.35 (1.16–1.66)	<0.001
**Urea** (mg/dL), median (IQR)	63 (39–102)	72.000 (51–100)	NS
**Creatinine** (mg/dL), median (IQR)	1.26 (0.87–2.16)	1.700 (1.125–2.645)	<0.01
**Total bilirubin** (mg/dL), median (IQR)	0.74 (0.43–1.24)	1.38 (0.635–2.48)	<0.001
**PaFi**, mean (SD) (mmHg)	292.5 (124.766)	228.3 (130.664)	<0.001
**SaFi**, mean (SD)	350 (117.39)	290 (109.66)	<0.01
**Lactate** (mmol/L), median (IQR)	2.00 (1.4–3.3)	4.70 (2.9–7.15)	<0.001
**Initial CRP** (mg/dL), median (IQR)	15.1 (6.4–26.53)	18.2 (7.825–28)	<0.05
**Initial PCT** (ng/mL), median (IQR)	1.84 (0.47–9.77)	27.415 (8.93–69.09)	<0.001
**proADM** (nmol/L), median (IQR)	2.795 (1.65–6.073)	10.000 (5.56–14.2)	<0.001
**Ferritin** (ng/mL), median (IQR)	566 (309.5–1813.5)	1619.5 (538–5925.75)	<0.001
**IgA** (mg/dL), median (IQR)	191 (113–260.5)	119 (80–175)	<0.001
**IgG** (mg/dL), median (IQR)	737.5 (525.5–999.5)	470 (353–699)	<0.001
**IgM** (mg/dL), median (IQR)	65.5 (38.75–104.25)	51 (26–9)	NS
**AKI**, n (%)	184 (13.9)	153 (44.1)	<0.001
**CRRT**, n (%)	77 (5.8)	91 (26.2)	<0.001
**HFNC**, n (%)	178 (13.5)	107 (30.8)	NS
**Dobutamine**, n (%)	54 (4.1)	77 (22.2)	<0.001
**Vasopressin**, n (%)	82 (6.2)	96 (27.7)	<0.001
**Left ventricular dysfunction**, n (%)	113 (8.5)	80 (23.0)	<0.001
**CRRT**, n (%)	144 (10.9)	100 (28.8)	<0.001
**Immunosuppression**, n (%)	506 (38.3)	169 (48.7)	<0.001
**IMV**, n (%)	338 (25.6)	155 (44.7)	<0.001
**IMV days**, median (IQR)	1 (0–13)	2 (0–14.5)	NS
**Length of stay**, (days) median (IQR)	14 (5–36)	11 (3–3)	<0.05
**Mortality**, n (%)	346 (26.2%)	174 (50.1%)	*p* < 0.001

**Table 4:** Multivariate logistic regression analysis identifying independent identify clinical and laboratory parameters independently associated with the level IL-6 > 15,000 pg/mL. β: beta regression coefficient, CRRT: continuous renal replacement therapy, IL-6: interleukin-6, IMV: invasive mechanical ventilation, INR: international normalized ratio, NS: not significant, PaFi: ratio of arterial oxygen partial pressure to fraction of inspired oxygen, PCT: procalcitonin, SE: standard error, SOFA: Sequential Organ Failure Assessment.

	Odds Ratio		SE	β	*p*-Value
SOFA	1.40	(1.10–1.82)	0.14	0.34	<0.05
PaFi	0.91	(0.75–1.10)	0.09	−0.10	NS
Lymphocyte count	0.60	(0.50–0.71)	0.09	−0.53	<0.001
Neutrophile count	0.53	(0.44–0.65)	0.09	−0.62	<0.001
Platelet count	1.23	(1.00–1.50)	0.10	0.20	NS
PCT	2.40	(2.00–2.86)	0.09	0.87	<0.001
INR	0.84	(0.69–0.97)	0.09	−0.17	NS
Lactate	1.28	(1.90–2.76)	0.09	0.83	<0.001
Vasoactive support	2.21	(1.37–3.60)	0.25	0.79	<0.01
Dobutamine	1.31	(0.63–2.76)	0.38	0.27	NS
Vasopressin	1.44	(0.85–2.44)	0.27	0.37	NS
Septic cardiomiopathy	1.10	(0.56–2.11)	0.33	0.10	NS
CRRT	0.80	(0.50–1.30)	0.24	−0.22	NS
Immunosuppression	0.92	(0.64–1.33)	0.18	−0.08	NS
IMV	0.61	(0.38–0.96)	0.23	−0.50	<0.05

**Table 5:** Clinical differences between survivors and non-survivors with IL-6 > 15,000 pg/mL. AKI: acute kidney injury, APACHE II: Acute Physiology and Chronic Health Evaluation II, CRP: C-reactive protein, CRRT: continuous renal replacement therapy, IgA: immunoglobulin A, IgG: immunoglobulin G, IgM: immunoglobulin M, IL-6: interleukin-6, IMV: invasive mechanical ventilation, INR: international normalized ratio, NS: rot significant, PaFi: ratio of arterial oxygen partial pressure to fraction of inspired oxygen, PCT: procalcitonin, proADM: proadrenomedullin, SaFi: ratio of arterial oxygen saturation to fraction of inspired oxygen, SD: standard deviation, SOFA: Sequential Organ Failure Assessment.

	Alive (n = 173)	Dead (n = 174)	*p*-Value
**Age** (years), mean (SD)	66 (18.993)	67 (14.717)	NS
**Gender** (female), n (%)	57 (32.9%)	63 (36.2%)	NS
**APACHE II**, mean (SD)	1 (8.601)	27 (8.867)	<0.001
**SOFA**, median (IQR)	8 (6–10)	12 (9–15)	<0.001
**Immunosuppression**, n (%)	58 (33.5%)	111 (63.8%)	<0.001
**Severe sepsis**, n (%)	39(22.5%)	17 (9.8%)	<0.01
**Septic shock**, n (%)	134 (77.5%)	157 (90.2%)	<0.01
**Leukocytes** (/mm^3^), median (IQR)	532 (1990–115.5)	272 (722.5–85.87)	<0.001
**Neutrophils** (/mm^3^), median (IQR)	400 (1200–850)	1200 (79.2–6125)	<0.001
**Lymphocytes** (/mm^3^), median (IQR)	200 (100–400)	200 (100–500)	NS
**Platelets** (×10^3^/L), median (IQR)	146 (83.7–207)	79.5 (35–157)	<0.001
**INR**, median (IQR)	1.29 (1.12–1.48)	1.44 (1.20–1.83)	<0.001
**Urea** (mg/dL), median (IQR)	62.5 (42.25–91.75)	81 (57–10)	<0.05
**Creatinine** (mg/dL), median (IQR)	1.525 (0.91–2.76)	1.81 (1.25–2.54)	NS
**Total bilirubin** (mg/dL), median (IQR)	1.3 (0.7–1.785)	1.46 (0.61–2.88)	NS
**PaFi**, mean (SD) (mmHg)	284.28 (126.5)	159.75 (121.84)	<0.001
**SaFi**, mean (SD)	345 (115.57)	250 (85.87)	<0.01
**Lactate** (mmol/L), median (IQR)	4.1 (2.65–6.25)	5.5 (3–8.475)	<0.001
**CRP** (mg/dL), median (IQR)	1 (7.27–27.43)	20 (8.64–29.08)	NS
**proADM** (nmol/L), median (IQR)	7.92 (4.86–10.82)	11.7 (7.865–18.85)	<0.001
**IL-6** (pg/mL), median (IQR)	62,773 (31,509–125,349)	98,341 (34,605.2–241,887)	<0.001
**Ferritin** (ng/mL), median (IQR)	790.5 (335.25–2077.75)	3470 (715.75 –7928.5)	<0.01
**IgA** (mg/dL), median (IQR)	123 (104–192)	116.5 (75.25–172.75)	NS
**IgG** (mg/dL), median (IQR)	572. (415–63)	428 (335.75–715)	NS
**IgM** (mg/dL), median (IQR)	54 (38–114)	45.5 (23.75–81.5)	NS
**PCT** (ng/mL), median (IQR)	24.05 (6.47–80)	29.68 (10.7)	NS
**AKI**, n (%)	65 (37.6%)	88 (50.6%)	<0.001
**CRRT**, n (%)	35 (20.2%)	65 (37.4%)	<0.001
**Dobutamine**, n (%)	31 (17.9%)	46 (26.4%)	NS
**Vasopressin**, n (%)	35 (20.2%)	61 (35.1%)	<0.01
**Left ventricular dysfunction**, n (%)	31 (17.9%)	49 (28.2%)	<0.05
**IMV**, n (%)	60 (34.7%)	95 (54.6%)	<0.001
**IMV days**, median (IQR)	2.5 (0–20.75)	2 (1–13.25)	NS
**Length of stay** (days), median (IQR)	19 (8–49)	4 (1–2)	<0.001

**Table 6:** Multivariate logistic regression analysis of factors independently associated with mortality in patients with IL-6 > 15,000 pg/mL. β: beta regression coefficient, CRP: C-reactive protein, IL-6: interleukin-6, INR: international normalized ratio, NS: not significant, PCT: procalcitonin, SE: standard error, SOFA: Sequential Organ Failure Assessment.

	Odds_Ratio		SE	β	*p*-Value
SOFA	3.41	(2.15–5.64)	0.245	1.23	<0.001
Immunosuppression	1.74	(2.94–11.42)	0.35	1.74	<0.001
PaFi	0.50	(0.30–0.81)	0.26	−0.69	<0.01
Platelet count	0.86	(0.60–1.21)	0.18	−0.15	NS
CRP	1.46	(1.02–2.10)	0.18	0.38	<0.05
PCT	0.71	(0.51–0.99)	0.17	−0.34	<0.05
INR	1.40	(0.91–2.26)	0.23	0.34	NS
Lactate	1.07	(0.75–1.54)	0.18	0.07	NS
Dobutamine	0.58	(0.25–1.31)	0.42	−0.56	NS

## Data Availability

The datasets used and/or analyzed during the current study are available from the corresponding author on reasonable request. To preserve as much as possible the identity of the patients, the data are not publicly available.

## Abbreviations

The following abbreviations are used in this manuscript:

AKI	Acute Kidney Injury
APACHE II	Acute Physiology and Chronic Health Evaluation II
ARDS	Acute Respiratory Distress Syndrome
BCP	Best Clinical Practice
CI	Confidence Interval
CRP	C-Reactive Protein
CRRT	Continuous Renal Replacement Therapy
HA	Hemoadsorption
ICU	Intensive Care Unit
Ig A	Immunoglobulin A
Ig G	Immunoglobulin G
Ig M	Immunoglobulin M
IL-6	Interleukin-6
IL-8	Interleukin-8
IL-10	Interleukin-10
IMV	Invasive Mechanical Ventilation
INR	International Normalized Ratio
IQR	Interquartile Range
ISC	In-Hospital Sepsis Code
LOS	Length of Stay
NS	Not Significant
OR	Odds Ratio
PaFiO_2_	PaO_2_/FiO_2_ Ratio (partial pressure of oxygen/fraction of inspired oxygen)
PCT	Procalcitonin
ROC	Receiver Operating Characteristic
SD	Standard Deviation
SE	Standard Error
SIRS	Systemic Inflammatory Response Syndrome
SOFA	Sequential Organ Failure Assessment
STROBE	Strengthening the Reporting of Observational Studies in Epidemiology
SWP	Standard Work Procedure
